# From Concept to Practice: Implementing a Knowledge-Driven Decision Support Platform for Sustainable Viticulture in Montenegro

**DOI:** 10.3390/s26092843

**Published:** 2026-05-01

**Authors:** Tamara Racković, Kruna Ratković, Marko Simeunović, Nataša Kovač, Christoph Menz, Helder Fraga, Aureliano C. Malheiro, António Fernandes, João A. Santos

**Affiliations:** 1 Faculty of Applied Sciences, University of Donja Gorica, Oktoih 1, 81000 Podgorica, Montenegro; tamara.rackovic@udg.edu.me (T.R.); kruna.ratkovic@udg.edu.me (K.R.); marko.simeunovic@udg.edu.me (M.S.); natasa.kovac@udg.edu.me (N.K.); 2 Potsdam Institute for Climate Impact Research (PIK) e.V., Telegrafenberg A 31, 14473 Potsdam, Germany; christoph.menz@pik-potsdam.de; 3 Centre for the Research and Technology of Agroenvironmental and Biological Sciences (CITAB), Inov4Agro, Universidade de Trás-os-Montes e Alto Douro (UTAD), Quinta de Prados, 5000-801 Vila Real, Portugal; hfraga@utad.pt (H.F.); amalheir@utad.pt (A.C.M.); acpf91@utad.pt (A.F.)

**Keywords:** decision support platform, viticulture, vineyard management, climate data, phenology, IoT, sustainability

## Abstract

Viticulture is highly vulnerable to weather variability and climate change. Growers increasingly face risks associated with extreme weather events, water scarcity, and emerging pests and diseases. To address these challenges, this study presents the development and implementation of the first operational digital decision support platform (DSP) tailored to Montenegrin vineyards within the MONTEVITIS project. The platform integrates IoT sensor data, national meteorological records and high-resolution global climate datasets to provide real-time monitoring and climate projections for vineyard management. The system was piloted in four vineyards representing diverse microclimatic and soil conditions of Montenegro. Key functionalities include phenology, irrigation and disease alerts supported by a user-friendly dashboard, map-based visualisation tools and data export functions. The pilot deployment demonstrated that combining heterogeneous data streams increases the reliability of outputs and enables timely, site-specific recommendations. Challenges identified during implementation include connectivity limitations, gaps in data and variable levels of digital expertise among growers; however, lessons learned point to the importance of continuous stakeholder engagement and institutional support for sustained use. The MONTEVITIS experience demonstrates how digital agriculture tools can bridge tradition and innovation in viticulture. By fostering collaboration between growers, researchers and policy makers, the platform enables adaptive strategies for climate resilience and sustainable vineyard management. Although the platform has been successfully deployed and tested under pilot conditions, a comprehensive long-term validation of its performance and impact on vineyard decision-making remains part of ongoing future work.

## 1. Introduction

Viticulture represents an important segment of Montenegrin agriculture, especially considering its role in regional wine production and the development of wine tourism [1]. The production of grapes and wine contributes noticeably to the agricultural economy and the broader alcohol sector, which carries measurable fiscal and economic effects in Montenegro [2]. Vine production has long been one of Montenegro’s key agricultural export sectors, accounting for approximately 10% of total agricultural exports [3]. In addition, viticulture and winemaking remain closely tied to Montenegro’s long-standing cultural and historical traditions, particularly within its established wine regions [1]. In 2024, vineyards in Montenegro covered a total area of 2553.3 ha, producing 16,869.3 tons of grapes, a figure that has been steadily declining in recent years [4]. There were 511 registered vineyards and 109 wineries in 2024 producing approximately 88.5 hl of wine, with Plantaže as the largest producer [3,5]. Plantaže are not only the biggest vineyard in Montenegro but also the largest single complex vineyard in Europe, spanning over 2300 ha, making up over 94.5% of the total Montenegrin production [6]. Viticulture in Montenegro is deeply embedded in the cultural identity of the country, with traditional winemaking techniques being passed down through generations and autochthonous grape varieties such as Vranac and Krstač, which are vastly planted, representing a unique genetic heritage [7].

The global wine sector has been facing important challenges due to global warming; specifically due to changes in climate involving a whole set of factors such as changes in the spatial patterns and temporal regimes of precipitation, temperature and humidity [8]. Rising temperatures induce long-term alterations in viticultural systems, potentially rendering entire regions unsuitable for grape cultivation [9]. Furthermore, common extreme climatic events can cause damage to a harvest or field. For example, shifts in precipitation patterns combined with temperature increase can change the frequency and intensity of droughts, potentially increasing the risk of grape stress, reducing yield and wine quality [10,11,12]. Additionally, Wu et al. [13] identified in the climate data analysis for 1961–2023 that more than 60% of Earth’s surface has been affected by heat waves. All of the mentioned factors represent essential variables in grape production, and any changes in them have a substantial impact on the final output [14]. These changes have particular impacts on yield, grape quality, wine quality and acidity [15]. Moreover, these changes hinder effective vine protection. Warmer winters favour the development of pests and diseases and accelerate vine phenological phases, leading to earlier harvests and potential challenges in meeting pre-harvest interval requirements [16]. Furthermore, climatic shifts not only impact grape yield and quality but also increase production costs due to higher irrigation needs, more frequent pest and disease treatments and others.

Alert systems supplied to viticulturists can prevent such problems by allowing them to apply appropriate adaptation measures. For example, timely pest or disease warnings could prevent significant yield losses and improve grape quality across multiple microclimates. Decrease in the yield trend and noticeable phenophases shifts require immediate action that enables the contribution of vinegrowers, policymakers, and researchers. Delivering this support requires an integrated system that gathers all three parties. Several countries have already developed dedicated decision-support platforms to address these challenges, including VitiMeteo [17,18] in Central Europe and mySense [19] in Portugal. VitiMeteo provides well-established forecasting models primarily focused on plant protection based on meteorological observations and biological disease-risk simulations, while mySense integrates IoT sensing, satellite observations, and UAV-based monitoring to support irrigation management and crop-status assessment at a regional scale. In contrast, the MONTEVITIS DSP was developed for operational deployment across Montenegro, where pronounced terrain heterogeneity and strong microclimatic variability over short spatial distances require dense in-field monitoring and localized alert-generation strategies, enabling stakeholder-oriented decision support adapted to region-specific environmental conditions. In contrast, the absence of a comparable platform in Montenegro limits the capacity of local viticulturists to respond effectively to these challenges. In the context of fast-paced climate change, the absence of a systematic framework that connects stakeholders and guides adaptation highlights key gaps in the implementation of precision viticulture. These gaps may limit the future development of viticulture and winemaking in Montenegro.

**Figure 1:**
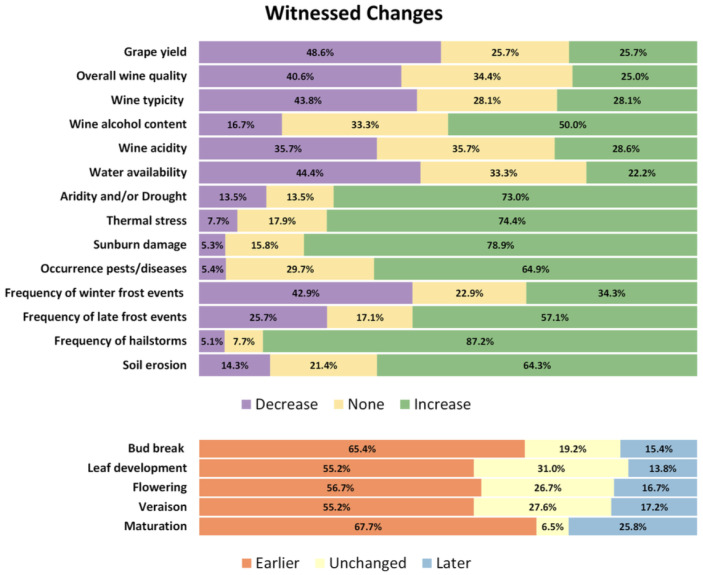
Survey results: witnessed changes by stakeholders.

The importance of developing such a framework was further confirmed through the analysis of a survey conducted during one of the workshops within the scope of the MONTEVITIS project [20]. The questionnaire aimed to explore perceptions of climate change and its potential effects on viticulture by professionals and stakeholders in Montenegro. Respondents were carefully selected based on their direct or indirect engagement with the sector, including viticulture, enology and related research activities. According to the questionnaire results presented in Figure 1, it is evident that the viticulture sector is already experiencing the impacts of climate change. The majority of responses align with theoretical expectations, indicating perceived decreases in grape yield, wine quality and water availability, alongside increases in frequency and intensity of extreme weather events and temperature-related damage. Among responses, the effects associated with rising temperatures and climatic extremes were the most consistently recognised by participants.

The survey analysis reinforces the need for adaptation measures in Montenegro. The usage of a decision platform is already an adaptation measure since it allows planning from the short to the long term. Such a platform would mostly help winegrowers to better understand local climate dynamics, assess potential risks and adopt strategies to safeguard the sector’s long-term sustainability. By integrating data, knowledge and tools, it could serve as a bridge between scientific research and on-the-ground decision making, promoting more resilience.

A conceptual preview of such a framework was developed within the scope of the ongoing Horizon Europe MONTEVITIS [20,21,22] project and introduced in our previous paper [23] as the first structured decision support platform (DSP) for viticulture in Montenegro. It aims at providing timely, localised viticultural scientific decision support based on real-time weather observations, historical climate data and climate projections in high resolution. Since Montenegro is a country with a very small area of 13,812 km^2^ [24], yet complex topography with several microclimates [25] and a sparse weather station network, it is important to include multiple point-sources. The sources proposed in this context are IoT sensor data, weather stations, the gridded future climate data CHELSA [26] (Climatologies at High Resolution for Earth’s Land Surface Areas) and observational data E-OBS [27]. The MONTEVITIS platform addresses the current fragmentation of agro-meteorological data sources by integrating information from multiple observational and modelling datasets into a unified decision-support framework.

To address these gaps, we proposed an integrated DSP structured around three main architectural layers: (i) Data Integration Layer combining local sensor data with external datasets; (ii) Decision Support Module using statistical and model-based approaches for prediction; and (iii) Interface Layer that presents results in a user-friendly format. Although thoroughly designed, this initial DSP model remained theoretical, and its functionality and impact under real-world conditions were yet to be validated. Without implementation, the continuous connection between the three parties that take an interest in viticulture and whose collaboration is one of the baselines for an efficient strategy, planning cannot be sustained. Rapid weather changes due to global warming and its significant consequences in the viticulture sector serve as a predominant motivation for prompt and actionable attempts to put this theoretical concept into action.

The DSP was established and implemented within the MONTEVITIS project, building upon the conceptual framework proposed in our previous work. It represents the first operational decision support system developed for viticulture in Montenegro. The platform introduces an integrated system architecture, organised around three functional pillars, that connects winegrowers, researchers and policy institutions through a shared data and decision support environment. As part of this framework, vineyard-level IoT weather stations were deployed at representative vineyard sites, establishing the first sensor-based microclimatic monitoring infrastructure for viticulture in Montenegro. These observations are integrated with national meteorological records and climate datasets derived from global repositories. The system harmonises these heterogeneous data sources within a single analytical framework adapted to the small spatial scale and strong microclimatic variability of Montenegro. This implementation establishes an integrated monitoring and decision support infrastructure for viticulture in Montenegro, combining vineyard-level sensor observations, national climate data and global climate datasets while enabling structured data exchange among stakeholders.

This study has three main objectives: (i) to demonstrate the platform’s technical functionality; (ii) to assess the platform’s dashboard design and role-specific outputs; (iii) to examine the potential role of the DSP in supporting climate-informed decision-making in Montenegrin viticulture. By addressing these objectives, the study provides empirical insights into the platform’s capabilities and lays the groundwork for its broader adoption across viticultural regions facing similar climatic challenges.

The paper is organised as follows. Section 2 focuses on the stakeholders and describes the characteristics of the partners’ vineyards. Section 3 presents the platform deployment, structured around three main pillars. Section 4 explains how decision support is delivered through alert systems for phenology, irrigation, and disease management, with additional functionality for yield simulation. Section 5 discusses the challenges encountered and lessons learned during the project, while Section 6 outlines future work and the platform’s potential for scalability.

## 2. Pilot Sites and Stakeholders

**Figure 2:**
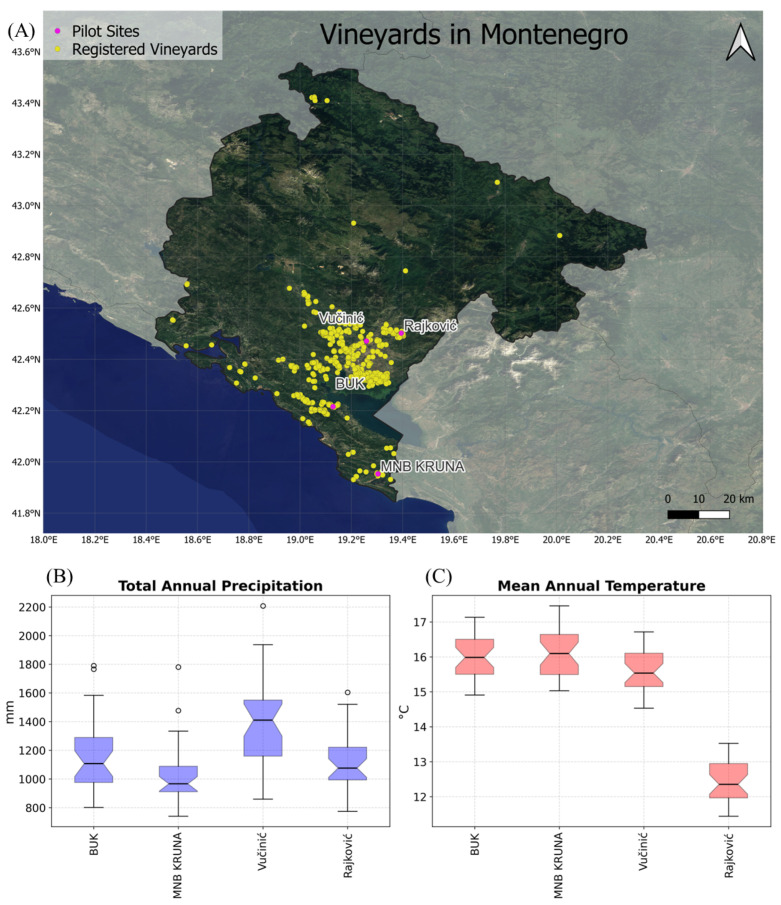
(**A**) Location of vineyards in Montenegro (available on Geoportal from Montenegro [32]) and location of Project MONTEVITIS pilot sites. Distribution of annual mean temperature and precipitation for 1981–2010 from EOBS: (**B**) mean annual average temperature and (**C**) total annual precipitation from EOBS [33].

Registered vineyards in Montenegro are distributed across a highly heterogeneous landscape, encompassing Mediterranean coastal and karst zones, sub-Mediterranean lowlands, and cooler mountainous regions. This geographical and climatological diversity results in markedly different grape growing conditions, climate impacts, and management requirements across vineyards. As a consequence, effective decision support in this context requires site-specific analysis and adaptation measures rather than uniform recommendations. Within this framework, four partner vineyards were incorporated as pilot sites to capture this variability and evaluate the platform’s capacity to support parcel-level, climate-informed decision-making. The selected family-operated vineyards are Vučinić, Rajković, BUK, and MNB KRUNA [28,29,30]. These four wineries are distributed across the central and southern regions of Montenegro, primarily within the Skadar Lake basin. The locations of the pilot sites and the rest of the cadastral registered vineyards [31] are represented in Figure 2, alongside climate normals for 1981–2010 from EOBS—mean annual average temperature and total annual precipitation. Mean annual temperature (normally distributed in all groups) differed significantly among stations (one-way ANOVA, *p* < 0.001); BUK did not differ from MNB KRUNA (Tukey HSD, *p* = 0.8854) or Vučinić (*p* = 0.0671). Total annual precipitation (non-normal in BUK and MNB KRUNA) also differed among stations (Kruskal–Wallis, *p* < 0.001); only BUK and Rajković did not differ (Mann–Whitney, *p* = 0.784460).

The selected pilot sites differ in vineyard size and grape varieties, introducing further dimensions of variability for evaluating site-specific impacts and management strategies. The range of terrain, microclimates and grape varieties shown in Table 1 illustrates the diversity of Montenegrin viticultural settings, supporting the platform to deliver site-specific functionalities and recommendations tailored to heterogeneous vineyard conditions across the country.

MNB KRUNA is located in Briska gora nearby Ulcinj in the south of Montenegro, with approximately 1.12 ha covered in grape vines. Rajković vineyards are nestled on the outskirts of Duklja in the mountain Kuči, located northwest of Podgorica. Accordingly, Rajković vineyards are the pilot site of the greatest elevation of 455 m [28]. BUK is a vineyard in the Crmnica area, specifically Bukovik village in the Skadar Lake basin, with 7500 grapevines [30]. Winery Vučinić is settled in the area of Rogami, near Podgorica, and it cultivates a large number of grape varieties. The diversity of locations, from the warm Mediterranean coastal–karst conditions of Briska Gora to the cooler, more humid sub-Mediterranean mountain climate of Kuči, and the terraced Mediterranean hillsides of Crmnica, ensures that the platform captures climatic variability across vineyards. These different conditions lead to distinct climate impacts on grape development, water stress and disease risk.

Autochthonous varieties Vranac and Kratošija are cultivated across all sites, representing a cornerstone of Montenegrin viticultural heritage. Alongside these, international varieties such as Cabernet Sauvignon, Chardonnay, and Marselan introduce additional diversity, highlighting the importance of considering varietal-specific responses within the platform. All the wineries have a long family tradition of vinegrowing passed down through generations.

These smaller family-run businesses, which represent the foundation of Montenegrin viticulture, could benefit from the DSP tools when it comes to optimisation of vineyard management on a parcel-by-parcel basis. Beyond the implemented pilot sites, additional users are anticipated, including large-scale producers such as Plantaže, which could benefit from coordinated production management under changing environmental conditions. This is particularly important as Plantaže is the predominant wine producer in Montenegro and one of the largest in the Balkans, focusing on both domestic and foreign wine markets.

While winegrowers represent the primary end users of the platform, additional user groups are anticipated, particularly researchers and institutional stakeholders. Researchers can utilise the continuously growing database for both academic and applied studies, including the analysis of long-term climatic trends, phenological shifts, and the development of data-driven approaches to grape variety sustainability. The platform is designed to support extensibility, enabling researchers to integrate new datasets, analytical methods, and modelling components, thereby expanding its functionality beyond the initial decision-support use cases. In this context, it may also serve as a centralised repository at the Centre for Climate Change, Natural Resources and Energy at UDG, facilitating the systematic integration of Montenegrin climatic observations with global climate datasets [34].

Another key stakeholder and end user is the Ministry of Agriculture, Forestry, and Water Management, operating at the national policy level. By integrating localised vineyard-level observations with regional and global climatic datasets, the platform provides a structured evidence base to support strategic planning and targeted policy interventions. Within this framework, researchers contribute scientific analyses of anticipated climate impacts and adaptation options, while winegrowers supply high-resolution field data and operational feedback. This bidirectional exchange allows winegrowers to ground regulatory and support requests in scientifically supported evidence, and enables policymakers to design climate-responsive agricultural policies and assistance programs based on empirically validated, multi-source information.

## 3. Platform Deployment

**Figure 3:**
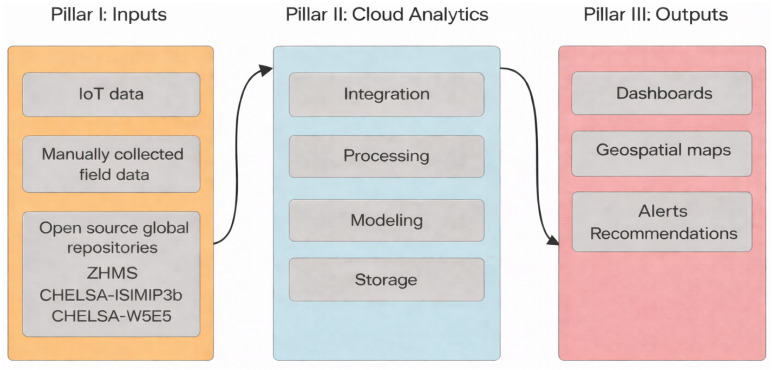
Data Flow Scheme.

The DSP presented in this study is implemented as a three-pillar architecture comprising data acquisition (Pillar I), cloud-based analytics and modelling (Pillar II), and a decision support delivery layer (Pillar III), which translates analytical outputs into actionable information for heterogeneous stakeholder groups. The global and local data path to the dashboard is represented in the data flow diagram in Figure 3.

While the three pillars are described sequentially, their functionality is inherently interlinked, with data acquisition and cloud analytics continuously informing the decision support layer, which operationalises these processes into spatially and temporally contextualised guidance for end users.

For the purpose of data acquisition, multiple data sources are employed, including IoT systems, field-collected data, weather stations and global repositories. The following paragraph focuses specifically on IoT-based data. Multiple sensors are installed at four project partners’ vineyard sites as IoT nodes, which operate as local in-field weather stations. There are several types of sensors that are collecting different data at high frequencies. The availability of particular sensor types across the vineyards is described in Table 2.

IoT sensors (WS3) record temperature, air humidity, wind speed, direction and strength, air pressure and precipitation. The sensors capture these readings at a 30 min interval, providing 48 daily measurements. This enables complete near real-time insight into weather conditions locally for timely decision making. Furthermore, CO_2_ sensor measures carbon concentration fluctuations that directly impact photosynthesis, resulting in physiological plant changes and phenology shifts [35]. A capacitive sensor measures relative humidity. This parameter directly affects vine health as high humidity supports fungal diseases (downy mildew, powdery mildew and *Botrytis cinerea*) and grape bursting [36,37]. The LTR390 is a digital sensor that detects UV-A (315–400 nm) or UV-B (280–315 nm) light. In viticulture it helps track solar UV exposure, which is important for plant growth, disease risk assessment, and sunburn prevention on grapes [38]. All data gathered through sensors are collected in IoT nodes via a wireless network. An overview of the sensors’ key characteristics is presented in Table 3.

**Figure 4:**
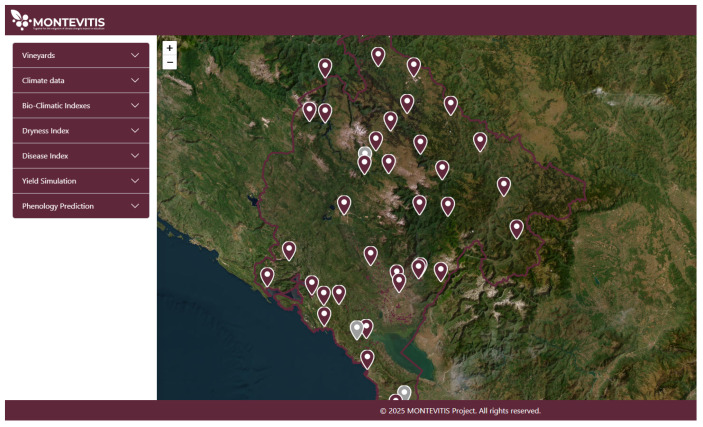
Integrated Network of National Meteorological and Vineyard IoT Stations.

Although IoT-based localized weather stations provide high-resolution, real-time data, certain limitations in data availability, such as missing values or deviations caused by periodic sensor failures, have been observed. To address these constraints, and to provide a more dense weather station network, data from national weather stations operated by the Montenegrin Institute for Hydrology and Seismology (ZHMS) is integrated [39]. Initially, the project included four weather stations located in Podgorica, Cetinje, Bar, and Ulcinj; however, since 2026, the platform has been receiving data from the complete network of ZHMS weather stations. IoT vineyard stations are used as the primary source of localized meteorological observations. In cases where IoT measurements are unavailable due to temporary sensor failures or missing values, the system automatically substitutes the corresponding observations with data from the nearest available national meteorological station operated by ZHMS. Additionally, for vineyards without installed IoT sensors, the platform automatically assigns meteorological inputs from the nearest ZHMS station based on the digital vineyard atlas, while informing the end user about the distance between the selected station and the vineyard location. A comprehensive overview of all available Vineyard IoT and national meteorological stations is provided within the platform, including real-time information on their operational status. This functionality is illustrated in Figure 4.

**Figure 5:**
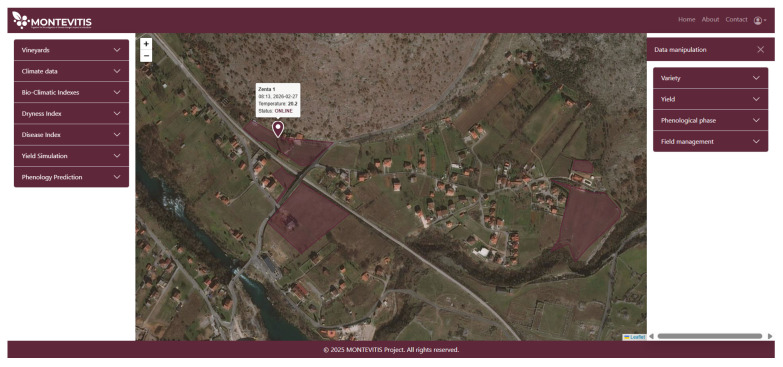
Initial Dashboard View with Vineyard Parcel Mapping.

To support evidence-based policy making and the generation of timely decision-support alerts, the platform integrates multiple data streams directly provided by viticulturists. The platform is designed to ensure simplified and user-friendly data input for viticulturists, as illustrated in Figure 5. For the purpose of detailed crop monitoring and robust model development, the occurrence of phenological events is systematically recorded through the platform interface. Users can upload georeferenced photographic documentation of the current phenological stage, accompanied by its corresponding phenological code according to the BBCH scale (Leaf development 11–29, Inflorescence emergence 53–57, Flowering 60–69, Fruit development 71–79, Ripening of berries 81–89, Senescence 91–99), together with the observation date, grape variety, and parcel identifier [40]. These records are aggregated into a structured historical phenological database, which serves as a foundation for the calibration and forecasting of phenological phase models.

Beyond phenological monitoring, the platform facilitates informed vineyard management by enabling the systematic recording of field operations. Documented management practices include treatment type (e.g., winter pruning of grapevines, removal of vine canes, basic fertilisation, herbicide application), date of application, and the associated grape variety and parcel. In addition, viticulturists report annual yield by variety, as well as detailed planting characteristics, including row spacing and intra-row planting distance. These variables collectively support the optimisation of yield and grape quality through improved data-driven management.

Additionally, upon authentication, users are provided with a geospatial overview of Montenegro displaying all pilot vineyard parcels, spatially referenced using cadastral geographic coordinates (Figure 5). This spatial framework establishes a location-based structure for linking vineyard management records to specific parcels. In addition to pilot sites, the platform incorporates registered vineyards from the Geoportal of the Cadastre and State Property Administration of Montenegro [32], thereby extending the spatial coverage and enabling parcel-level data association within a broader regional analytical context. The spatial representation of the registered vineyards, as displayed on the platform’s geospatial interface, constitutes a unique digital Montenegrin viticultural atlas.

To ensure a broader analytical perspective and to incorporate global data repositories beyond the IoT-derived measurements, the platform also integrates climate datasets, including both observational records and simulated projections. The Observations module within the Climate Data section is based on the CHELSA-W5E5 v1.0 dataset [41], which represents W5E5 reanalysis data (WFDE5 merged with ERA5) downscaled using the CHELSA v2.0 framework to approximately 1 km spatial resolution. The dataset provides gridded historical climate fields (1981–2010) and serves as the observational baseline within the platform. Detailed methodological information on W5E5 bias adjustment and the CHELSA downscaling procedure is available in Lange (2019) [42], Cucchi et al. (2020) [43], and Karger et al. (2021) [44].

For long-term decision making based on climate projections, the CHELSA downscaling framework [44,45] was used to produce a high-resolution database (hereinafter referred to as CHELSA-ISIMIP3b). The employed dataset on the platform originates from bias-adjusted climate projections of the Inter-Sectoral Impact Model Intercomparison Project (ISIMIP3b; 0.5° ≈50 km resolution) [46,47], derived from the Coupled Model Intercomparison Project Phase 6 (CMIP6) [48,49]. Daily outputs from nine GCMs were processed for both historical (1981–2014) and future (2015–2100) periods. The selected GCMs were: CanESM5, CNRM-CM6-1, CNRM-ESM2-1, EC-Earth3, IPSL-CM6A-LR, MIROC6, MPI-155 ESM1-2-LR, MRI-ESM2-0, and UKESM1-0-LL. To explore different future scenarios, three Shared Socio-Economic Pathways (SSPs) were processed for the future period: SSP1-2.6, representing a sustainability-oriented future (lower GHG emissions); SSP3-7.0, a moderately pessimistic trajectory; and SSP5-8.5, a high-emission, fossil-fuel-intensive scenario [50]. The dataset includes daily minimum, mean, and maximum temperature and precipitation downscaled to 30 arc-second resolution (approximately 1 km). These data sets are integrated into the platform’s modelling component, allowing users to visualise both historical baselines and projected future conditions at vineyard scale.

Daily data served as input for the calculation of bioclimatic indices, calculated for each GCM, scenario and year. Indices related to viticulture reported in the literature as relevant for decision making were computed: total annual precipitation [51]; growing season precipitation [52]; annual average temperature [53]; growing season temperature [51]; growing degree days for a base of 10° [54]; Winkler index [55]; biologically effective degree days [56]; hydrothermal index of Branas; Huglin index [57]; cool night index [57]; dryness index [57] and De Martonne Index [58]. Additionally, temperature and precipitation extremes indices were computed to display the future potential risk of extreme events [59]. These indices are relevant in the context of decision-making; for example, the Winkler index classifies wine-growing regions and wine types based on the accumulation of temperature [55]. Another example is the average growing season temperature, which allows for selecting the most suitable grape varieties [60].

**Figure 6:**
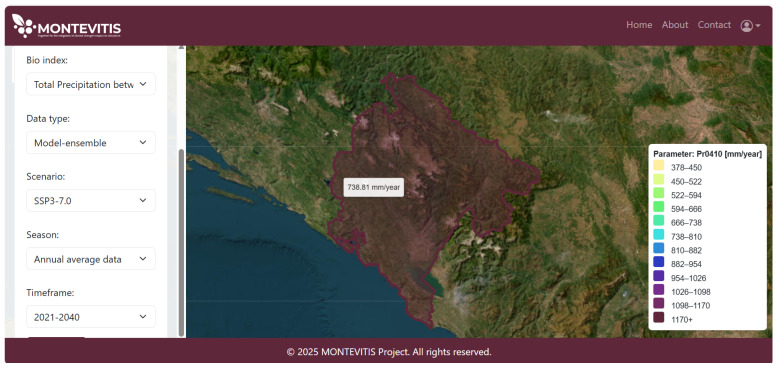
Geospatial visualisation of CHELSA-based climate normal (2021–2040) for total precipitation (April–October) under the SSP3 scenario.

For the bioclimatic indices (annual time steps), the nine-member ensemble mean was calculated for each scenario for periods of 20 or 30 years (e.g., 2041–2060, 2041–2070), for the time range 1981–2100. Climate normals of bioclimatic indices are subsequently operationalised within the decision support delivery layer (Pillar III) through geospatial representations on the platform, enabling interpretation of long-term climate change. In contrast, the annual bioclimatic indices and daily data are available upon request. These two groups are not directly accessible on the platform, as this data requires higher computing capacity and expertise to interpret. To ensure the platform remains user-friendly, only multi-model ensemble means of bioclimatic climate normals are directly accessible. For example, the platform representation of the climate normal (2021–2040) of total precipitation between April and October under SSP3 is presented in Figure 6.

Building on the heterogeneous data streams described in Pillar I, the cloud-based analytics layer (Pillar II) operates on preprocessed, validated, and harmonized datasets to perform data integration, aggregation, and modeling, thereby transforming curated observations and climate projections into decision-relevant indicators. Within this module, IoT data are verified and complemented through gap-filling procedures using ZHMS meteorological records to ensure input reliability and consistency. ZHMS observations, originally collected at a 30 min temporal resolution, are subsequently aggregated to derive daily summary statistics.

The platform architecture is centred on a cloud-based infrastructure that supports data ingestion, processing, and storage. This modular, cloud-deployed design ensures scalability and facilitates the efficient integration of heterogeneous datasets. Data from IoT and ZHMS sources are transmitted to the cloud through a standardised RESTful API [61]. IoT nodes communicate with the cloud in real time via GSM or LoRa [62] wireless uplinks, with data formatted in JSON and delivered through secure HTTPS requests. Both IoT and ZHMS data are automatically ingested and stored in a centralised database. Once ingested, the data is processed within the cloud-based processing layer. Additional aggregation and derivation steps are performed, such as the computation of hourly (1 h) and daily (24 h) precipitation totals, as well as minimum, maximum, and mean temperature values. Weather data can be aggregated at hourly, daily, or annual temporal scales, depending on end-user requirements, and are presented through user-friendly graphical visualisations for selected time periods. Although system-level performance metrics such as low-latency response times, strict uptime guarantees, or zero data-loss rates are commonly used in real-time control systems, their relevance in the context of climate-driven decision support platforms is inherently limited. The MONTEVITIS DSP operates on aggregated temporal scales (e.g., 30-min sampling intervals and daily model updates), where minor delays in data transmission or processing do not significantly affect the validity of generated indicators or alerts. Similarly, short-term data gaps caused by IoT sensor interruptions do not critically impact system outputs, as the platform integrates multiple data sources and implements automatic gap-filling procedures using national meteorological observations (ZHMS), thereby preserving temporal continuity and analytical robustness.

From an operational perspective, system performance is therefore better characterised in terms of data availability, redundancy, and robustness rather than strict latency or loss minimisation. The distributed IoT infrastructure provides near real-time environmental monitoring, while the cloud-based architecture ensures scalable data ingestion, processing, and storage. The combination of heterogeneous data streams and fallback mechanisms enables stable platform operation under real-world conditions, even in the presence of connectivity limitations and partial data loss, which are typical for agricultural IoT deployments.

The MONTEVITIS platform implements a distributed data storage and retention architecture designed to ensure long-term data availability, integrity, and system resilience. All collected data are retained permanently, as no deletion policy is applied, enabling longitudinal climate analyses, model calibration, and retrospective evaluation of vineyard management practices. Raw high-resolution IoT observations are stored on a primary server in their original 30-min temporal resolution, while aggregated and processed datasets (e.g., hourly and daily summaries and derived indicators) are stored on a secondary geographically dislocated server, providing an additional redundancy layer. Both servers are subject to periodic backup procedures, with full system backups performed on a 15-day cycle and optional incremental backups between cycles to reduce potential data-loss windows. This architecture enables recovery of datasets in the event of hardware failure, data corruption, or connectivity disruptions and provides baseline disaster-recovery capability appropriate for agricultural decision-support applications. Additional safeguards include secure HTTPS-based RESTful API communication and structured validation during data ingestion to ensure consistency and reliability of incoming data streams.

Within the modelling layer, deterministic bioclimatic indices, such as the Goidanich [63] and Dryness indices [64], are operationalised as part of a continuous analytical pipeline to generate risk-based indicators and support automated alert generation. The Goidanich index is computed using hourly temperature and precipitation data to derive a cumulative risk metric reflecting the progressive development of disease-favourable conditions. The Dryness index relies primarily on hourly precipitation measurements to characterise short-term soil moisture deficits and surface wetness conditions relevant to vineyard water stress assessment. These are examined in detail through application-specific use cases in subsequent sections.

Pillar III constitutes the decision support delivery layer that operationalises the analytical outputs of Pillar II by providing role-specific access to climate indicators, modelled risk metrics, and historical trends. Rather than serving solely as a visualisation interface, this layer supports situational awareness, spatial reasoning, and coordination among heterogeneous stakeholder groups, thereby facilitating operational planning and longer-term adaptation strategies. Detailed examples of alert-driven and management-oriented workflows are presented in the subsequent use-case section.

The delivery layer is designed to present complex, multi-source data in a user-centred and accessible format, ensuring interpretability across stakeholder groups with varying levels of technical expertise. The outputs illustrated in Figure 3 represent end-user products of the platform, highlighting the role of interface customisation in translating analytical and processed data into actionable information. Within this framework, the platform provides geospatial visualisations of gridded climate normals and historical and future climate projections derived from the CHELSA dataset, enabling spatially explicit interpretation of long-term precipitation and temperature patterns across vineyard regions.

**Figure 7:**
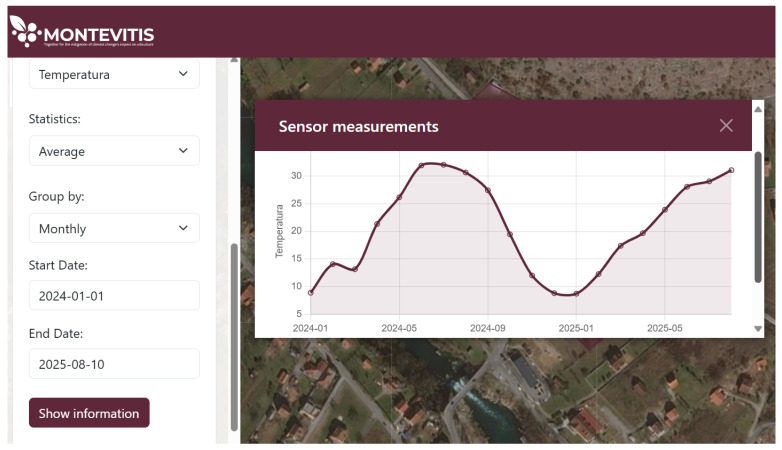
Average monthly temperature for the period 1 January 2024–10 August 2025.

At the vineyard scale, the dashboard exposes real-time and historical IoT observations alongside parcel-level information, including vineyard boundaries, parcel number and area by grape variety, registered phenological records, and vineyard management data. Users can explore temporal variability through descriptive statistics (minimum, maximum, and mean values) and multiple temporal aggregation levels (daily, monthly, and annual), enabling comparative assessment of recent conditions and longer-term trends. A representative example of average monthly and daily temperature dynamics for the Vučinić vineyard is shown in Figure 7, illustrating how localised climate information can support site-specific decision-making. Additionally, phenological records can be provided in PDF or XLSX formats for user-defined time frames to facilitate documentation and further analysis.

## 4. Integrated Alert-Based Decision Support

This section presents real-world applications of the platform across different stages of vineyard management, operated by different stakeholders. Building on the data representation presented in Section 3, this section examines the implementation of scenario-specific alerts to support strategic and evidence-based decision making. Each use case scenario focuses on a specific functionality—phenology estimations, irrigation recommendations and disease alerts—demonstrating the platform’s capacity to address key operational and management challenges in vineyard practice. The presented use cases serve as operational validation scenarios, demonstrating how the proposed framework performs under real-world vineyard conditions.

### 4.1. Irrigation Alert

Prolonged summer droughts, combined with high temperatures, frequently exceeding 40 °C, substantially increase the risk of vine water stress, leading to reductions in yield and grape quality [65]. Water management is therefore a critical adaptation measure, particularly in periods preceding the onset of stress, as delayed or insufficient water supply can result in irreversible physiological damage. The platform provides irrigation recommendations based on the integration of real-time local climate observations with historical patterns. In parallel, the increasing occurrence of intense summer precipitation events can temporarily reduce irrigation demand, enabling more efficient allocation of water resources across parcels. In the Montenegrin context, such optimisation also supports the maintenance of strategic water reserves for wildfire response during the dry season. Irrigation assessment approach is formulated as a Dryness Index (DI), which is computed as a recursive daily soil water balance:

$$DI_{t}=\min\left(200,\;DI_{t-1}+P_{t}-T_{v}-E_{s}\right),$$

where $DI_{t}$ represents soil water availability on day *t*, initialized with a soil water reserve of 200 mm, $P_{t}$ is daily precipitation, $T_{v}$ is vine transpiration, and $E_{s}$ is soil evaporation. The evapotranspiration components are estimated as

$$T_{v}=k_{1}ETP_{t},$$

$$E_{s}=\left(1-k_{1}\right)\;ETP_{t}\cdot \min\left(\frac{P_{t}}{5},1\right),$$

where $k_{1}$ is a seasonal crop coefficient. Daily potential evapotranspiration $ETP_{t}$ was calculated using the Thornthwaite formulation based on mean temperature, photoperiod, and the annual heat index coefficient [66]. The threshold value DI=50 was selected as an operational alert level consistent with threshold ranges commonly used in viticultural Dryness Index-based water availability assessment frameworks [67]. In the MONTEVITIS platform, this value is therefore applied as an operational indicator of increasing dryness conditions requiring irrigation attention rather than as a site-specific physiological threshold.

**Figure 8:**
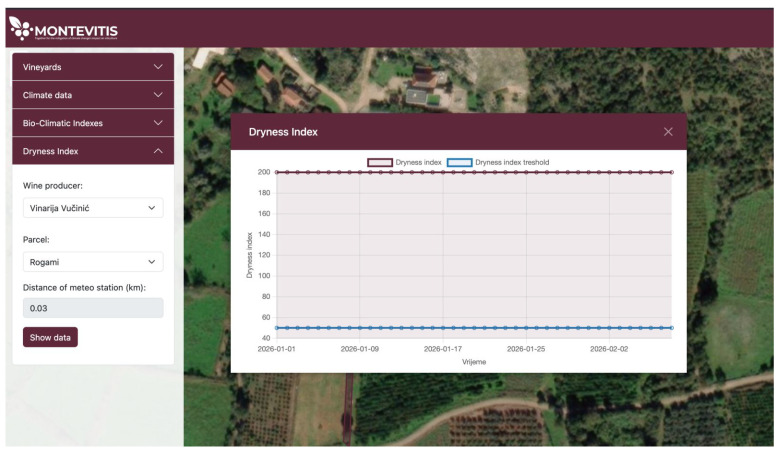
Annual profile of the daily Dryness Index (DI).

The Dryness Index is presented on an annual basis and, under the current winter-season conditions, does not exceed the defined threshold, as illustrated in Figure 8. It is expected that it will exhibit a seasonal pattern, with values declining below the defined threshold during the summer period, thereby triggering the irrigation alert.

Recent DSS approaches based on IoT data have focused on the real-time monitoring and prediction of the crop coefficient (Kc) [68]. The crop coefficient reflects crop water demand, with lower values indicating reduced demand and higher values indicating increased water requirements. While Kc-based methods estimate crop water demand directly, the approach proposed in this study supports irrigation decisions based on meteorological information. These approaches are therefore related and complementary.

### 4.2. Disease Alert

Downy mildew is one of the main pathogens found in grapevine in the Mediterranean region, and can cause yield losses up to 75–100% if left untreated [69]. Timely intervention against the disease could result in long-lasting beneficial outcomes. Disease risk alerts for downy mildew are implemented using the standard Goidanich index as the core decision model. The system operates in two sequential phases [70]. The initial phase represents the onset of primary infection and is triggered when the so-called ‘3–10 rule’ is satisfied, i.e., when cumulative precipitation exceeds 10 mm within a 24-h period, mean air temperature is above 10 °C, and vine shoot length exceeds 10 cm. Once these conditions are met, the model transitions to a daily risk accumulation phase, in which infection risk is computed as a function of relative humidity and temperature using the Goidanich index scale. The Goidanich index (GI) was computed as a cumulative mildew development indicator updated daily after fulfillment of the 3–10 infection rule:

$$GI_{t}=\min\left(100,\;GI_{t-1}+f\left(T_{t},RH_{t}\right)\right),$$

where $GI_{t}$ represents cumulative infection risk on day *t*, $RH_{t}$ is daily relative humidity, and $f(T_{t},RH_{t})$ represents temperature relative humidity development coefficients obtained from the standard Goidanich table for *Plasmopara viticola* infection risk estimation [71]. This formulation follows established agrometeorological disease forecasting approaches widely used in viticultural decision support systems. Meteorological inputs used for Goidanich index computation were aggregated to daily temporal resolution prior to model application. Daily mean air temperature was derived from minimum and maximum temperature values, daily accumulated precipitation was used for activation of the 3–10 infection rule, and daily mean relative humidity was used to determine mildew development increments from the Goidanich table. For IoT stations providing 30-min observations, temperature and relative humidity were averaged to daily values, while precipitation was accumulated to daily totals before index calculation.

**Figure 9:**
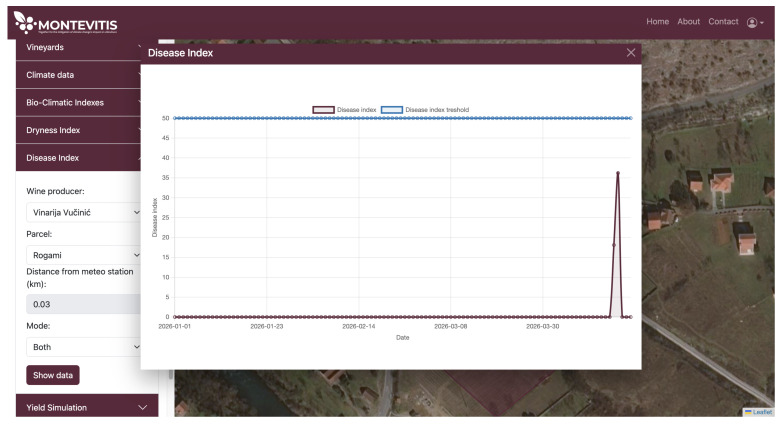
Annual profile of the daily Disease Index.

When the cumulative risk exceeds a predefined threshold of 50%, the platform automatically issues an alert to the viticulturist through the decision support interface, accompanied by a recommendation for timely treatment application. The disease alert output is presented on an annual basis and is currently based on input data for 2026. The first registered favourable conditions for downy mildew development are shown in Figure 9; however, the calculated risk index remains below the alarm threshold of 50 and therefore does not trigger a warning signal. Under expected seasonal temperature increases and precipitation patterns, an accumulation of disease risk is expected in upcoming seasons.

The system operates in automatic, manual, and hybrid modes, enabling the integration of site-specific management data directly into the modelling framework. Among the management inputs, recorded spraying events are explicitly incorporated, resulting in the reset of the simulated disease risk index to zero. This coupling between management actions and model dynamics allows adaptive updating of risk estimations. In the absence of recorded management data, a rule-based assumption is applied whereby treatment is considered to occur once the predefined risk threshold is exceeded.

**Figure 10:**
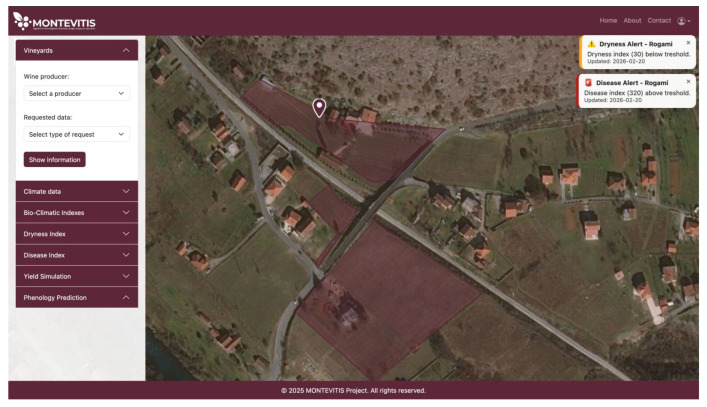
Dryness and Disease alerts for Vučinić vinery.

Both disease and irrigation alerts are not limited to graphical outputs or detailed trend analysis. Instead, the platform provides simplified real-time notifications to ensure timely and user-friendly decision support for different stakeholder groups, as presented in Figure 10.

### 4.3. Phenology Alert

Phenological modelling supports the timing of key vineyard management operations, including pruning, fertilisation, pest control, canopy management, and harvest preparation, all of which are closely linked to specific growth stages. Climate-driven shifts in phenological timing, particularly for bud burst and flowering, increase the risk of mismatches between management actions and plant development, as well as exposure to frost events that can substantially reduce seasonal yield [72]. Timely phenological alerts therefore assist growers in planning labour-intensive operations, such as canopy interventions on large parcels, and in mitigating risks associated with unexpected early-season cold events.

**Figure 11:**
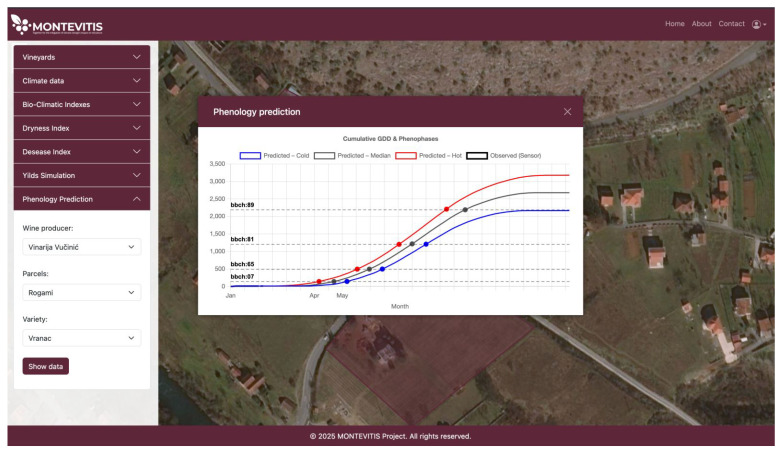
GDD estimates for Vranac variety, derived from historical data from E-OBS v 32, using daily percentile distributions.

The DSP phenology estimations are based on the Growing Degree Days (GDD) approach [73]. When a threshold accumulation of active temperatures (above 10 °C) is reached, typically the phenological phase occurs. During the MONTEIVITS project, we have gathered the phenology data of Vranac and other grape varieties from pilot sites. For Vranac, the respective GDD phenological phases are: Bud burst beginning BBCH: 07 (Bud burst beginning)-139 °C; BBCH: 65 (Full flowering-50% of flowers open)-492; BBCH: 81 (Beginning of ripening-veraison)-1203; BBCH: 89 (Berries ripe for harvest)-2188. In this sense, the platform registers the site temperature from the sensor, calculates the cumulative GDD (since the first of January), and estimates the following growing degree days for the dates after the last sensor reading until the end of the year. In Figure 11, we present estimates derived from historical data from E-OBS v 32, using daily percentile distributions. Daily percentiles 25%, 50%, 75% correspond to the estimates of Cold, Median and Hot. This approach is dynamic: since each day there are new sensor readings, the estimations of phenological dates become more reliable due to the use of more measured data and less forecasted data. This represents, to our knowledge, the first documented implementation of GDD-based phenological modelling for the autochthonous variety Vranac.

### 4.4. Yield Simulation

Yield simulation was conducted using a process-based grapevine growth model specifically parameterized and calibrated for the autochthonous Montenegrin cultivar Vranac. The model represents vine growth by simulating biomass accumulation as a function of intercepted photosynthetically active radiation and radiation use efficiency, with dynamic allocation of assimilates to reproductive organs following anthesis. Final yield is defined as the simulated fruit dry biomass at physiological maturity.

The model explicitly accounts for environmental constraints, particularly water limitation, through the implementation of the Fraction of Transpirable Soil Water (FTSW) [74,75]. This variable regulates biomass production under declining soil moisture conditions, reducing growth rates when soil water content falls below cultivar-specific critical thresholds. In addition, vineyard management practices are incorporated as model input variables. Certain practices, such as pruning and canopy trimming, directly affect leaf area development and total biomass accumulation, whereas others, such as irrigation, enhance soil water availability and mitigate water stress effects. By integrating climatic variability, soil properties, and management interventions, the model establishes a mechanistic linkage between environmental drivers and final yield formation.

Furthermore, the DSP enables simulation of yield responses under different climate projections, to provide a broader overview that possibly suggests mitigation and adaptation strategies. The platform allows the selection of one of nine Global Climate Models, under three Shared Socioeconomic Pathways, enabling the explicit consideration of climate model uncertainty. Simulations are performed under both present (1985–2014) and future climates (2030–2070), allowing comparison of expected yield trends across time horizons.

**Figure 12:**
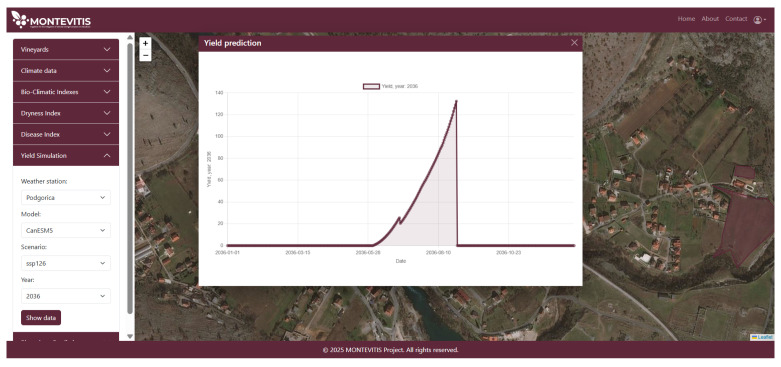
Yield simulation under CanESM5 model SSP1 scenario for 2036.

By integrating site selection, climate model choice, and management inputs into a unified modelling framework, the DSP translates complex climate–growth interactions into quantitative yield projections for Vranac, supporting climate-resilient decision-making in Montenegrin viticulture. An example of the yield simulation output for the year 2036 under the SSP1-2.6 scenario is presented in Figure 12, illustrating the projected fruit biomass response for the selected site and climate model configuration.

Although alert-driven use cases constitute the primary operational interface for winegrowers, the DSP is implemented as a unified system environment shared across all stakeholder groups. Customization is achieved through differences in data resolution, analytical depth, and modes of representation that align with the distinct decision-making scopes of each user category. Winegrowers primarily interact with high-resolution, real-time alerts and simplified visual outputs to support rapid, site-specific interventions. Researchers, within the same interface, access time-series visualizations, geospatial analyses, and structured phenological datasets to examine spatiotemporal trends and to develop and validate predictive models. Policymakers engage with aggregated regional indicators and long-term climate projections that support strategic planning, policy formulation, and the design of targeted support instruments. By providing a common set of standardized indicators and reference datasets at different levels of analytical complexity, the platform harmonizes access to and interpretation of decision-relevant information and facilitates coordinated, evidence-based responses across the viticulture value chain.

## 5. Challenges and Lessons Learned

The real-world deployment of the MONTEVITIS platform across heterogeneous vineyard environments revealed a set of technical, operational and institutional challenges that extend beyond system architecture and model implementation. These challenges provided important insights into the conditions required for the long-term sustainability and transferability of climate-informed decision support systems in agricultural practice. The result represents years of coordinated work, combining technical innovation with field experience.

The operation of distributed IoT nodes in field conditions resulted in recurring gaps and inconsistencies in the local readings, primarily associated with connectivity constraints, power supply interruptions and occasional sensor malfunctions. Addressing these disruptions required the implementation of a dedicated preprocessing workflow to validate, harmonize and reconstruct the affected time series prior to their integration into the platform, as described in Section 3. A key lesson learned was that the reliability of alert-based decision support outputs depends fundamentally on the continuity and comparability of the data streams. The integration of heterogeneous sources, including local sensors, national meteorological stations and gridded climate, necessitated systematic data quality management as a foundational component of system operation.

Beyond technical performance, platform utilization was strongly influenced by user engagement, digital literacy and institutional context. Winegrowers were required to contribute field-level observations, such as phenological stages and basic management actions, through the platform interface. In practice, variability in the timeliness and consistency of these inputs introduced additional uncertainty into model-based estimates and alerts. Targeted training and continued technical support emerged as essential measures for improving data completeness and ensuring sustained platform use. This experience highlights that decision support systems in traditional agricultural sectors are socio-technical systems in which model performance and data quality are closely coupled with user practices and institutional capacity.

At the governance level, questions related to data ownership, access rights, and the role of public institutions in maintaining and scaling the platform underscored the need for formal institutional engagement. Without long-term support structures, digital platforms risk remaining project-based prototypes rather than operational components of agricultural decision-making frameworks.

Ongoing technical support represents a core requirement for the long-term operation of the platform. Beyond user assistance, this includes the continuous monitoring, maintenance and restoration of IoT nodes in cases of power supply interruptions, connectivity outages, or hardware-related failures. The timely recovery of these components is essential for preserving the continuity and reliability of local measurement records and, by extension, the validity of the associated decision support outputs.

These operational requirements highlight the need for a strategically planned financial sustainability framework. While initial development and deployment were supported through Horizon project funding, the platform is envisioned as a long-term component of the national agricultural support infrastructure. A subscription-based service model, in which users contribute through periodic service fees, represents one potential mechanism for covering operational and maintenance costs. However, the long-term objective is to minimize financial barriers to access and to position the platform as a public-support infrastructure that promotes broad adoption and contributes to the environmental, economic, and cultural sustainability of viticulture.

Achieving this objective requires formal institutional engagement with the Ministry to establish governance structures, budgetary pathways, and coordinated deployment strategies, complemented by collaboration with research institutions, industry stakeholders, and agricultural agencies. The lessons learned and partnerships established through the pilot deployment provide a foundation for extending the platform to wider regional contexts and to additional agricultural systems, thereby supporting both functional scalability and long-term institutional integration.

## 6. Future Work and Scalability

While the present study demonstrates the technical implementation and pilot deployment of the MONTEVITIS DSP, a comprehensive validation of its long-term performance and its impact on vineyard decision-making was beyond the scope of this work. The current results primarily illustrate the operational feasibility of the system and its ability to integrate heterogeneous data streams into a functional decision support environment. A structured qualitative evaluation involving stakeholder interviews, usability studies, and systematic assessment of user-related factors such as satisfaction, trust, and perceived usefulness is planned as part of the post-project phase. Since the platform constitutes the final deliverable of the MONTEVITS project and was fully deployed only at the end of the project period, stakeholders have only recently gained access to the complete system, and additional time is required to support a meaningful and representative evaluation. Future research will therefore focus on systematic validation through longer observational periods, expanded vineyard participation and the evaluation of platform-supported decisions against observed management outcomes.

Platform sustainability implies continuous improvement of the modelling components deployed within the system, including the integration of machine learning approaches. Reported phenological stages provided by winegrowers are accompanied by field photographs and subsequently reviewed by project researchers to ensure consistency with the BBCH scale; observations that cannot be confirmed from the visual evidence are excluded from further use. However, this process could be streamlined by introducing ML-based methods capable of automatically detecting BBCH growth stages from image data [76]. In the interim, targeted field training for end users is recommended to improve the accuracy and consistency of manually recorded phenological observations and to support the quality of the resulting training datasets. In parallel, ML models can be deployed to support irrigation-related decision-making by estimating vine water stress and irrigation demand based on agroclimatic and physiological inputs [77]. Grapevine yield prediction, particularly during advanced phenological stages and in the pre-harvest period, represents a critical component of effective vineyard management, as it supports planning, resource allocation, and market-oriented decision-making. The integration of ML-based yield prediction models that relate historical yield records to agroclimatic patterns can therefore be considered a key future extension of the platform [78]. In addition, supplementary IoT measurements, such as sap flow sensors [79], can be integrated to enhance the estimation of vine water status and irrigation demand.

Two additional types of predictive alerts can be considered as future extensions of the MONTEVITIS platform, depending on data availability: immediate alerts operating on short time scales (minutes to hours), such as notifications of approaching storms, and near-term warnings operating on longer time scales (days to weeks), including indications of adverse weather conditions, drought development, or frost risk. Near-term warnings are particularly relevant for routine vineyard management, as they support operational planning and resource allocation over several days and enable the implementation of adaptation measures such as frost protection or irrigation scheduling. Forecast based alert components are already commonly implemented in operational viticultural decision-support systems and represent a logical next step for further development of the MONTEVITIS platform. Their integration could be achieved either by connecting to freely available global forecasting services (e.g., open meteorological APIs [80]) or through a direct data link with ZHMS, enabling the use of national operational forecasts. Incorporating such a forecasting component would allow end users to access long term climate projections together with mid and short term weather forecasts within a single interface, further supporting decision making processes.

Building on recent advances in the application of unmanned aerial vehicles (UAVs) in viticulture, the integration of such data sources can be considered as a potential extension of the platform [81]. In this context, UAV-based observations can further enrich the system by enabling detailed spatial and temporal monitoring of vineyard conditions. For example, spatial heterogeneity in canopy growth or differences in vine water stress within a single parcel can be identified. In parallel, satellite-based Earth observation data can complement this information by providing consistent, large-scale coverage and regular temporal sampling, enabling the assessment of seasonal trends and regional patterns in vegetation condition and climatic stress [82]. The fusion of these spatial products with point-based IoT measurements supports multi-scale analysis and zone-specific decision support outputs.

One potential direction for future research is the integration of the proposed system with certification and labelling systems commonly used in viticulture, including organic and sustainable standards and the GLOBALG.A.P. framework for Good Agricultural Practices [83]. In addition, geographical indication schemes (GI/PGI/DO) could be considered to support the protection of geographic origin and product heritage. Moreover, integration with these platforms would enhance transparency, traceability and market competitiveness. Furthermore, multilingual support should be considered, as Montenegro has become a destination for international migration, and a diverse user base may include non-native speakers among potential platform users. The core function of the platform is the timely delivery of alerts and decision support, one remaining technical challenge concerns reliable message delivery. In case the winegrower is not connected to the internet it is still important to receive an alert, therefore SMS-based alert delivery should be considered as a complementary communication channel in situations of limited internet connectivity.

The MONTEVITIS methodology, which integrates local IoT measurements, regional weather station data, gridded climate products, and vineyard-specific observations such as phenology and yield, is not limited to deployment in Montenegro. Scalability to other Mediterranean viticultural zones is enabled by substituting national meteorological networks and reference datasets with equivalent regional services, while preserving the same parcel-level decision support logic. This requires the recalibration of phenological models and alert thresholds to locally dominant grape varieties and management practices, supporting deployment in coastal, island, and mountainous regions characterised by fragmented vineyards and strong microclimatic gradients.

Beyond viticulture, the platform architecture can be extended to other crop systems by adapting the decision support logic to crop-specific phenological stages and weather-driven management processes, including olive groves, citrus orchards, and cereal production [84]. This transferability is enabled by substituting grapevine-specific phenological models and bioclimatic indices with crop-specific indicators, threshold values, and management-relevant alert logic while preserving the same data ingestion, validation, and decision support pipeline. In practice, this allows the system to support applications such as drought stress monitoring in rainfed cereals, heat and frost risk assessment in citrus orchards, or phenology-driven pest management in perennial tree crops, thereby positioning the platform as a modular decision support framework adaptable across multiple climate-sensitive agricultural sectors.

## 7. Conclusions

This study demonstrates the practical feasibility and added value of a knowledge-driven digital decision-support platform for climate-resilient and sustainable viticulture in data-scarce and topographically complex regions. By integrating local IoT-based observations, national meteorological records, gridded climate datasets, and vineyard-level phenological and management inputs, the MONTEVITIS platform delivers parcel-specific indicators and operational alerts that address critical dimensions of vineyard management, including phenological timing, irrigation planning, and disease risk mitigation.

The pilot deployment across four heterogeneous vineyards provided a real-world deployment of the proposed DSP approach, confirming that multi-source data can substantially improve the reliability and spatial relevance of decision-support outputs. The implemented use cases illustrate how climate-informed models can be operationalized within a unified system environment and adapted to diverse vineyard sizes, terrain conditions, and management practices, thereby supporting timely, evidence-based decisions under increasing climate variability.

Beyond technical performance, the findings highlight that the long-term effectiveness and sustainability of digital decision-support systems are fundamentally shaped by stakeholder engagement and institutional integration. Continuous input of field-level observations by winegrowers, combined with analytical refinement by researchers and strategic use of aggregated indicators by policymakers, establishes a feedback loop that strengthens model accuracy and aligns operational vineyard management with longer-term adaptation and policy planning. This socio-technical interaction emerged as a critical enabler for sustained platform adoption and for embedding the DSP within broader agricultural support frameworks.

Taken together, the MONTEVITIS experience provides a transferable framework for the development of climate-informed decision-support systems in Mediterranean and temperate agricultural contexts. By coupling technological innovation with participatory governance and iterative feedback mechanisms, the platform contributes to strengthening the climate resilience, environmental sustainability and long-term viability of viticultural systems and other climate-sensitive agricultural sectors. Nevertheless, the present study focuses primarily on demonstrating the technical implementation and operational feasibility of the platform, while a systematic evaluation of its long-term performance and decision-support effectiveness across multiple growing seasons remains a subject of future research.

## Figures and Tables

**Table 1:** Vineyard description.

Vineyard	Vučinić	Rajković	BUK	MNB KRUNA
**Region**	Central	Central	Southern	Southern
**Municipality**	Podgorica	Podgorica	Bar	Ulcinj
**Microclimate**	Sub-Mediterranean river-valley basin	Sub-Mediterranean mountain (transition to continental)	Mediterranean hillside-terraced	Warm Mediterranean coastal–karst
**Number of parcels**	36	18	20	8
**Total area (ha)**	2.00	0.96	1.22	1.12
**Grape varieties**	Vranac, Marselan, Cabernet Sauvignon, Chardonnay, Malvasija, Kratošija,	Vranac, Cabernet Sauvignon, Chardonnay, Kratošija, Čubrica, Smederevka	Vranac, Cabernet Sauvignon, Muscat Ottonel, Marselan, Chardonnay	Vranac, Kratošija

**Table 2:** IoT devices across vineyards.

Data Collection	Vučinić	Rajković	BUK	MNB KRUNA
WS3	✓	✓	✓	✓
CO_2_ sensor	✓	✓	✓	✓
Capacitive sensor for humidity	✓	✓	✓	✓
UV sensor				✓

**Table 3:** Sensors’ particularities.

Sensors	Parameters	Units	Time Series/Freq	Data Transmission
WS3	Temperature	°C	30 min	Wireless uplink GSM/LoRa
	Air humidity	%		
	Wind speed	m/s		
	Air pressure	hPa		
	Precipitation	mm		
CO_2_ sensor	CO_2_ concentration	ppm	30 min	Wireless uplink
	Board temperature	°C		
Capacitive sensor for humidity	Relative humidity	%	30 min	Wireless uplink
LTR390-UV sensor	UV radiation	μW/cm^2^	30 min	Wireless uplink

## Data Availability

The data presented in this study are available on request from the corresponding author.

## References

[1] Perović N., Ristanović B., Pavićević Đ., Koval V. (2024). Analysis of the promotion of small wine producers in wine regions of Montenegro and the perspective of wine tourism in cooperation. Agric. For..

[2] Mugoša A., Kovačević M., Čizmović M. (2024). National Study-Montenegro, Economics of Alcohol and Alcohol Taxation.

[3] Analiza Poslovanja Crnogorske Privrede u 2024. Godini, Privredna Komora Crne Gore. https://komora.me/wp-content/uploads/2025/01/cg-privreda-2024-w.pdf.

[4] Monstat. https://www.monstat.org/cg/.

[5] Plantaže. https://www.plantaze.com/.

[6] Pajović-Šćepanović R., Krstić M., Savković S., Raičević D., Popović T. (2016). Wine quality in Montenegro. Agric. For./Poljoprivreda I ŠUmarstvo.

[7] Maraš V., Popović T., Gazivoda A., Raićević J., Kodžulović V., Mugoša M., Šućur S. (2015). Origin and characterization of Montenegrin grapevine varieties. VITIS-J. Grapevine Res..

[8] Thornton P., Ericksen P., Herrero M., Challinor A. (2014). Climate variability and vulnerability to climate change: A review. Glob. Change Biol..

[9] Hannah L., Roehrdanz P.R., Ikegami M., Shepard A.V., Shaw M.R., Tabor G., Zhi L., Marquet P.A., Hijmans R.J. (2013). Climate change, wine, and conservation. Proc. Natl. Acad. Sci. USA.

[10] Gambetta G.A., Herrera J.C., Dayer S., Feng Q., Hochberg U., Castellarin S.D. (2020). The physiology of drought stress in grapevine: Towards an integrative definition of drought tolerance. J. Exp. Bot..

[11] Venios X., Korkas E., Nisiotou A., Banilas G. (2020). Grapevine responses to heat stress and global warming. Plants.

[12] Keller M. (2023). Climate Change Impacts on Vineyards in Warm and Dry Areas: Challenges and Opportunities: From the ASEV Climate Change Symposium Part 1—Viticulture. Am. J. Enol. Vitic..

[13] Wu S., Luo M., Lau G.N.C., Zhang W., Wang L., Liu Z., Lin L., Wang Y., Ge E., Li J. (2025). Rapid flips between warm and cold extremes in a warming world. Nat. Commun..

[14] Santillán D., Garrote L., Iglesias A., Sotes V. (2020). Climate change risks and adaptation: New indicators for Mediterranean viticulture. Mitig. Adapt. Strateg. Glob. Change.

[15] Baltazar M., Castro I., Gonçalves B. (2025). Adaptation to Climate Change in Viticulture: The Role of Varietal Selection—A Review. Plants.

[16] Santos J.A., Fraga H., Malheiro A.C., Moutinho-Pereira J., Dinis L.T., Correia C., Moriondo M., Leolini L., Dibari C., Costafreda-Aumedes S. (2020). A Review of the Potential Climate Change Impacts and Adaptation Options for European Viticulture. Appl. Sci..

[17] Staatliches Weinbauinstitut Freiburg (2026). VitiMeteo Baden-Württemberg: Forecasting System for Plant Protection in Viticulture. https://www.vitimeteo-bw.de/vitimeteo/default/index.

[18] Dubuis P.H., Bleyer G., Krause R., Viret O., Fabre A., Werder M., Naef A., Breuer M., Gindro K. (2019). VitiMeteo and Agrometeo: Two platforms for plant protection management based on an international collaboration. Bio Web Conf..

[19] Morais R., Silva N., Mendes J., Adão T., Pádua L., López-Riquelme J.A., Pavón-Pulido N., Sousa J.J., Peres E. (2019). mySense: A comprehensive data management environment to improve precision agriculture practices. Comput. Electron. Agric..

[20] Montevitis. https://montevitis.eu/montevitis-project/.

[21] Horizon Europe: The EU’s Key Funding Programme for Research and Innovation. https://research-and-innovation.ec.europa.eu/funding/funding-opportunities/funding-programmes-and-open-calls/horizon-europe_en.

[22] Montevitis Platform. http://montevitisserver2.udg.edu.me:5001/pocetna.

[23] Simeunović M., Ratković K., Kovač N., Racković T., Fernandes A. (2025). A Knowledge-Driven Framework for a Decision Support Platform in Sustainable Viticulture: Integrating Climate Data and Supporting Stakeholder Collaboration. Sustainability.

[24] Danube Facts and Figures, March 2010. https://www.icpdr.org/sites/default/files/Montenegro%20Facts%20Figures%20FINAL.pdf.

[25] Burić D., Ducić V., Mihajlović J. (2014). The climate of Montenegro: Modificators and types-part two. Bull. Serbian Geogr. Soc..

[26] CHELSA—Climatologies at High Resolution for the Earth’s Land Surface Areas. https://chelsa-climate.org/.

[27] In-Situ Gridded Observations for Europe. https://cds.climate.copernicus.eu/datasets/insitu-gridded-observations-europe?tab=overview.

[28] Vinarija Rajović. https://vinarijarajkovic.com/.

[29] Vinarija Vučinić. https://www.vinarijavucinic.com/.

[30] Vinarija Buk. https://bukwinery.me/me/.

[31] Real Estate Administration, Government of Montenegro (2025). Real Estate Administration of Montenegro—Cadastral and Property Register. Official Cadastral Authority Responsible for Real Estate Cadastre and Parcel Data in Montenegro. https://www.gov.me/uzn.

[32] Geoportal CG—Geoportal of Cadastre and State Property Administration of Montenegro. Interactive Web Map and Metadata Portal. https://www.geoportal.co.me/geoportal/geoportal_eng.html.

[33] Cornes R.C., Van Der Schrier G., Van Den Besselaar E.J., Jones P.D. (2018). An ensemble version of the E-OBS temperature and precipitation data sets. J. Geophys. Res. Atmos..

[34] Centar za Klimatske Promjene, Prirodne Resurse i Energiju. https://ccc.udg.edu.me/me/.

[35] Tang Y., Zhou W., Du Y. (2023). Effects of temperature, precipitation, and CO_2_ on plant phenology in China: A circular regression approach. Forests.

[36] Portela F., Sousa J.J., Araújo-Paredes C., Peres E., Morais R., Pádua L. (2025). Monitoring the Progression of Downy Mildew on Vineyards Using Multi-Temporal Unmanned Aerial Vehicle Multispectral Data. Agronomy.

[37] Ramteke S.D., Urkude V., Parhe S.D., Bhagwat S.R. (2017). Berry cracking; its causes and remedies in grapes-a review. Trends Biosci..

[38] Gambetta J.M., Holzapfel B.P., Stoll M., Friedel M. (2021). Sunburn in grapes: A review. Front. Plant Sci..

[39] Zavod za Hidrometeorologiju i Seizmologiju. https://www.meteo.co.me/.

[40] Lorenz D., Eichhorn K., Bleiholder H., Klose R., Meier U., Weber E. (1995). Growth Stages of the Grapevine: Phenological growth stages of the grapevine (*Vitis vinifera* L. ssp. vinifera)—Codes and descriptions according to the extended BBCH scale. Aust. J. Grape Wine Res..

[41] Karger D.N., Lange S., Hari C., Reyer C.P., Zimmermann N.E. (2021). CHELSA-W5E5 v1.0: W5E5 v1.0 Downscaled with CHELSA v2.0.

[42] Lange S. (2019). WFDE5 Over Land Merged with ERA5 Over the Ocean (W5E5), Version 1.0.

[43] Cucchi M., Weedon G.P., Amici A., Bellouin N., Lange S., Müller Schmied H., Hersbach H., Buontempo C. (2020). WFDE5: Bias-adjusted ERA5 reanalysis data for impact studies. Earth Syst. Sci. Data.

[44] Karger D.N., Wilson A.M., Mahony C., Zimmermann N.E., Jetz W. (2021). Global daily 1 km land surface precipitation based on cloud cover-informed downscaling. Sci. Data.

[45] Karger D.N., Conrad O., Böhner J., Kawohl T., Kreft H., Soria-Auza R.W., Zimmermann N.E., Linder H.P., Kessler M. (2017). Climatologies at high resolution for the earth’s land surface areas. Sci. Data.

[46] Lange S. (2019). Trend-preserving bias adjustment and statistical downscaling with ISIMIP3BASD (v1.0). Geosci. Model Dev..

[47] Frieler K., Volkholz J., Lange S., Schewe J., Mengel M., del Rocío Rivas López M., Otto C., Reyer C.P., Karger D.N., Malle J.T. (2024). Scenario setup and forcing data for impact model evaluation and impact attribution within the third round of the Inter-Sectoral Impact Model Intercomparison Project (ISIMIP3a). Geosci. Model Dev..

[48] Eyring V., Bony S., Meehl G.A., Senior C.A., Stevens B., Stouffer R.J., Taylor K.E. (2016). Overview of the Coupled Model Intercomparison Project Phase 6 (CMIP6) experimental design and organization. Geosci. Model Dev..

[49] O’Neill B.C., Tebaldi C., van Vuuren D.P., Eyring V., Friedlingstein P., Hurtt G., Knutti R., Kriegler E., Lamarque J.F., Lowe J. (2016). The Scenario Model Intercomparison Project (ScenarioMIP) for CMIP6. Geosci. Model Dev..

[50] Meinshausen M., Nicholls Z.R., Lewis J., Gidden M.J., Vogel E., Freund M., Beyerle U., Gessner C., Nauels A., Bauer N. (2020). The shared socio-economic pathway (SSP) greenhouse gas concentrations and their extensions to 2500. Geosci. Model Dev..

[51] Bunting E.L., Wanyama D., Goodwin R., Weil N., Sabbatini P., Andresen J. (2021). Vitis vinifera production in Michigan: Factors and trends driving cultivation patterns. Front. Plant Sci..

[52] Adão F., Campos J.C., Santos J.A., Malheiro A.C., Fraga H. (2023). Relocation of bioclimatic suitability of Portuguese grapevine varieties under climate change scenarios. Front. Plant Sci..

[53] Schultz H.R., Jones G.V. (2010). Climate Induced Historic and Future Changes in Viticulture. J. Wine Res..

[54] Brun P., Zimmermann N.E., Hari C., Pellissier L., Karger D.N. (2022). Global climate-related predictors at kilometer resolution for the past and future. Earth Syst. Sci. Data.

[55] Amerine M.A., Winkler A.J. (1963). California Wine Grapes: Composition and Quality of Their Musts and Wines.

[56] Gladstones J. (2011). Wine, Terroir and Climate Change.

[57] Fonseca A., Cruz J., Fraga H., Andrade C., Valente J., Alves F., Neto A.C., Flores R., Santos J.A. (2024). Vineyard microclimatic zoning as a tool to promote sustainable viticulture under climate change. Sustainability.

[58] Claro A.M., Fonseca A., Fraga H., Santos J.A. (2023). Susceptibility of Iberia to extreme precipitation and aridity: A new high-resolution analysis over an extended historical period. Water.

[59] Schulzweida U., Quast R. Climate indices with CDO: Climate indices of daily temperature and precipitation extremes. Technical report, 2015. https://gitlab.earth.bsc.es/ces/cdo/raw/b4f0edf2d5c87630ed4c5ddee5a4992e3e08b06a/doc/cdo_eca.pdf.

[60] Jones G.V. (2018). The Climate Component of Terroir. Elements.

[61] Fielding R.T. (2000). Architectural Styles and the Design of Network-Based Software Architectures.

[62] Centenaro M., Vangelista L., Zanella A., Zorzi M. (2016). Long-range communications in unlicensed bands: The rising stars in the IoT and smart city scenarios. IEEE Wirel. Commun..

[63] Rossi V., Caffi T., Giosuè S., Bugiani R. (2008). A mechanistic model simulating primary infections of downy mildew in grapevine. Ecol. Model..

[64] Moriondo M., Jones G., Bois B., Dibari C., Ferrise R., Trombi G., Bindi M. (2013). Projected shifts of wine regions in response to climate change. Clim. Change.

[65] Chaves M.M., Santos T.P., Souza C.d., Ortuño M., Rodrigues M., Lopes C., Maroco J., Pereira J.S. (2007). Deficit irrigation in grapevine improves water-use efficiency while controlling vigour and production quality. Ann. Appl. Biol..

[66] Thornthwaite C.W. (1948). An approach toward a rational classification of climate. Geogr. Rev..

[67] Tonietto J., Carbonneau A. (2004). A multicriteria climatic classification system for grape-growing regions worldwide. Agric. For. Meteorol..

[68] Kocian A., Cela F., Carmassi G., Citti S., Malorgio F., Paganelli F., Chessa S., Milazzo P., Incrocci L. (2026). Combining dynamic Bayesian prediction of the crop coefficient with automated lysimetry for highly accurate water-use control. Comput. Electron. Agric..

[69] Maddalena G., Marone Fassolo E., Bianco P.A., Toffolatti S.L. (2023). Disease forecasting for the rational management of grapevine mildews in the Chianti Bio-District (Tuscany). Plants.

[70] Fernández-González M., Piña-Rey A., González-Fernández E., Aira M., Rodríguez-Rajo F. (2019). First assessment of Goidanich Index and aerobiological data for *Plasmopara viticola* infection risk management in north-west Spain. J. Agric. Sci..

[71] Pérez-Expósito J.P., Fernández-Caramés T.M., Fraga-Lamas P., Castedo L. (2017). VineSens: An eco-smart decision-support viticulture system. Sensors.

[72] Bucur G.M., Dejeu L. (2020). Researches on the frost resistance of grapevine with special regard to the Romanian viticulture. A review. Sci. Pap. Ser. B Hortic..

[73] Verdugo-Vásquez N., Pañitrur-De la Fuente C., Ortega-Farías S. (2017). Model development to predict phenological scale of table grapes (cvs. Thompson, Crimson and Superior Seedless and Red Globe) using growing degree days. Oeno ONE.

[74] Bindi M., Bellesi S., Orlandini S., Fibbi L., Moriondo M., Sinclair T. (2005). Influence of water deficit stress on leaf area development and transpiration of Sangiovese grapevines grown in pots. Am. J. Enol. Vitic..

[75] Tanner C., Sinclair T. (1983). Efficient water use in crop production: Research or research?. Limitations to Efficient Water Use in Crop Production.

[76] Schieck M., Krajsic P., Loos F., Hussein A., Franczyk B., Kozierkiewicz A., Pietranik M. (2023). Comparison of deep learning methods for grapevine growth stage recognition. Comput. Electron. Agric..

[77] Brillante L., Mathieu O., Lévêque J., Bois B. (2016). Ecophysiological modeling of grapevine water stress in burgundy terroirs by a machine-learning approach. Front. Plant Sci..

[78] Sirsat M.S., Mendes-Moreira J., Ferreira C., Cunha M. (2019). Machine Learning predictive model of grapevine yield based on agroclimatic patterns. Eng. Agric. Environ. Food.

[79] Mancha L.A., Uriarte D., Prieto M.d.H. (2021). Characterization of the transpiration of a vineyard under different irrigation strategies using sap flow sensors. Water.

[80] Meteoblue AG (2024). Meteoblue Weather and Climate Data Platform. https://www.meteoblue.com.

[81] Singh A.P., Yerudkar A., Mariani V., Iannelli L., Glielmo L. (2022). A bibliometric review of the use of unmanned aerial vehicles in precision agriculture and precision viticulture for sensing applications. Remote Sens..

[82] Pasqualotto N., D’Urso G., Bolognesi S.F., Belfiore O.R., Van Wittenberghe S., Delegido J., Pezzola A., Winschel C., Moreno J. (2019). Retrieval of evapotranspiration from sentinel-2: Comparison of vegetation indices, semi-empirical models and SNAP biophysical processor approach. Agronomy.

[83] Global Good Agricultural Practices. https://www.ecocert.com/en-US/certification-detail/sustainable-farming-ggp.

[84] Quiroga S., Iglesias A. (2009). A comparison of the climate risks of cereal, citrus, grapevine and olive production in Spain. Agric. Syst..

